# Energy materials: core/shell structural photoelectrodes assembled with quantum dots for solar cells

**DOI:** 10.3402/nano.v4i0.21080

**Published:** 2013-06-11

**Authors:** J. J. Tian, Q. F. Zhang, L. L. Zhang, R. Gao, L. F. Shen, S. G. Zhang, X. H. Qu, G. Z. Cao

**Affiliations:** 1 Advanced Material and Technology Institute, University of Science and Technology Beijing, Beijing, P.R. China; 2 Department of Materials and Engineering, University of Washington, Seattle, WA, USA

## Core-shell photoelectrodes for energy harvesting

Narrow-band-gap semiconductor quantum dots (QDs) are considered as next-generation sensitizers for solar cells because of their extraordinary optical and electrical properties in terms of tuneable band gap, high molar extinction coefficient, and large intrinsic dipole moment, which may facilitate charge separation in solar cells. Moreover, theoretical photovoltaic conversion efficiency of QDs-sensitized solar cells can reach up to 44% in view of multiple exciton generation (MEG).

**Figure:**
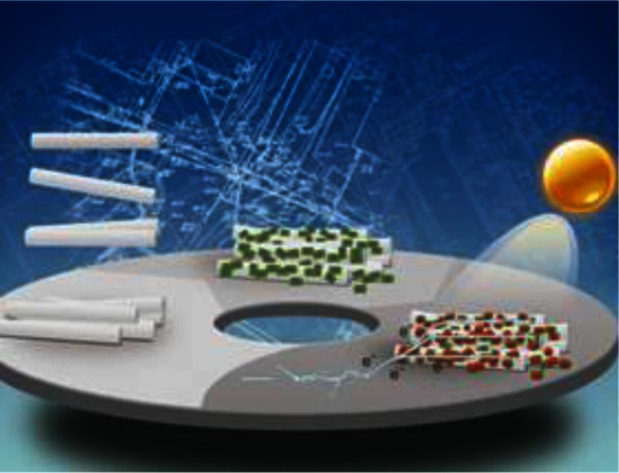


As we know, ZnO is considered as one of the optimal candidate photoelectrodes for quantum dot sensitized solar cells (QDSCs) due to its high electron mobility, suitable energy-band structure, and excellent physical properties. In addition, ZnO easily forms anisotropic structure such as nanowires, nanorods, and nanotubes, presenting unique electronic and optical properties. Furthermore, a photoelectrode film constructed with these nanostructures is helpful for the distribution of QDs. Tian and colleagues (1) at the University of Science and Technology Beijing and Cao and colleagues (1) at the University of Washington at Seattle have designed ZnO nanorods (NR) photoelectrodes for QDSCs. ZnO NRs serve as the backbone for direct electron transport in view of its single crystallinity. Nevertheless, the efficiency of QDSCs is poor and likely due to high surface charge recombination in ZnO. To improve the efficiency of QDSC, Tian and colleagues (1) prepared a photoelectrode made of nanocable core-shell structure of ZnO NRs coated with TiO_2_ nanosheets (NSs). Anatase TiO_2_ NSs with a thickness of ∼10 nm and a length of ∼100 nm are assembled onto the surface of ZnO NRs via a solvothermal method. The thin shell of TiO_2_ shows a remarkable effect on QDSCs by increasing the surface area of ZnO NRs to allow for adsorbing more QDs, which leads to high short current density. Also, the unique material prepared here has an energy barrier that hinders the electrons in the ZnO from being transferred to the electrolyte or QDs, and thus, reduces the charge recombination rate, increases electron lifetime, and enhances open voltage. In comparison with the case of ZnO NRs, the short-circuit current density, open-circuit voltage, fill factor, and charge recombination resistance of ZnO/TiO_2_ nanocable photoelectrode increase by 3, 44, 48 and 220%, respectively. As a result, a power conversion efficiency of 2.7% in QDSCs with a core-shell structural nanocable photoelectrode has been obtained, which is as much as 230% of that obtained for ZnO NR photoelectrode.
